# A Novel Paradigm to Design Personalized Derived Images of Art Paintings Using an Intelligent Emotional Analysis Model

**DOI:** 10.3389/fpsyg.2021.713545

**Published:** 2021-07-09

**Authors:** Yingjing Duan, Jie Zhang, Xiaoqing Gu

**Affiliations:** ^1^School of Design, Jiangnan University, Wuxi, China; ^2^School of Computer Science and Artificial Intelligence, Changzhou University, Changzhou, China

**Keywords:** artificial intelligence, emotional analysis, emotional features, art derivatives, design paradigm

## Abstract

With the development of artificial intelligence (AI), it is imperative to combine design methods with new technologies. From the perspective of the personalized design of derived images of art paintings, this study analyzes the new user demand generated by the current situation and background of personalized design, puts forward a new method of derivative design based on AI emotion analysis, verifies the feasibility of the new method by constructing a personalized design system of derived images of art paintings driven by facial emotion features, and explores the method of combining AI emotion recognition, emotion analysis, and personalized design. This study provides new ideas for the design of art derivatives for the future with massive personalized demand. Thinking and practicing from the perspective of the development of new technology will promote the change of design paradigms in the digital age.

## Introduction

In art derivative design, designers use modern design methods to popularize artistic connotations into the modern landscape by thinking, transforming, and designing after fully understanding the connotation of the derived original art. It enables derivatives to meet the spiritual needs of users by carrying a certain artistic connotation. Unlike ordinary products, the willingness of users to buy art derivatives is more out of a desire for the exhibit or works of art in the exhibit. Therefore, it is important for art derivatives to meet the emotional needs of users as much as possible. With the advent of the era of participatory consumption, an increasing number of derivative designs have begun to incorporate personalized factors; only carrying the artistic connotation of art derivatives cannot simply meet the emotional needs of users. The addition of personalized elements can help to realize the prominent and differentiated embodiment of the personal meaning of consumers, accordingly establishing the emotional connection between derivatives and users. Therefore, the combination of derivatives and personalization is an inevitable trend in the design of art derivatives. However, from the current situation of derivative design, the current product form is immutable, the design method is monotonous, and the cost to meet personalized design needs is high. Therefore, future derivative design has to not only meet the demand for the thousands of needs of people in quantity but also realize the meaning connection between individuals and products from the perspective of consumer psychology to be meaningfully accurate to the individual users.

With the rise of GPU parallel computing, especially the explosion of big data, the development of artificial intelligence (AI) technology has become increasingly faster. Among them, the emergence of deep learning is particularly important (Tseng and Ho, 2012; Al-Saffar et al., 2017; Kumar et al., 2019; Mccormack et al., 2020; Somasundaram et al., 2020; Hughes et al., 2021). Initially, deep learning was used in the field of automated speech recognition, which resulted in a significant increase in recognition rates (Krizhevsky et al., 2017). Then, the application of convolutional neural networks (CNNs) has achieved remarkable success in image recognition. Subsequently, generative adversarial nets (GAN) (Goodfellow et al., 2014) that emerged in 2014 was a huge success in areas such as image generation and style migration. In the field of design, deep learning has also been fruitful. For example, an automatic generation system of user portraits was built by Salminen et al. (2020) and An et al. (2018) to isolate behavioral and demographic segments for persona creation *via* aggregated user data. The generation model proposed by Wang et al. (2017) and Bharadhwaj et al. (2018) can be used for the construction of potential user portraits. The creative-stimulating models built by Chen et al. (2019) through semantic associations and the ability to design models for a rapidly built prototype interface introduced by the design software Figma help designers to build quick prototypes. The advancement of deep learning challenges the original design paradigm, and there are feasible ways to meet the needs of “thousands of needs” and “emotional connection.”

## The Process of Personalized Design of Artistic Image Derivatives Based on Emotional Analysis

**Figure 1:**
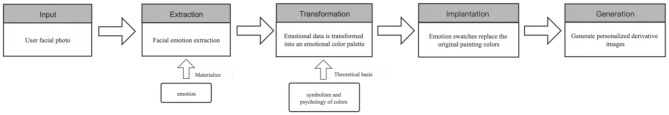
The design process based on emotion analysis.

Facing these problems, based on AI technology, this study constructs a new method of generating art derivative images driven by user emotional representation and forms a set of personalized design processes of art image derivatives based on emotion analysis. The process is divided into five main stages: input, extraction, transformation, implantation, and generation. Because emotion is abstract, in extracting features, this study chooses the figurative expression of emotion, i.e., facial expression, as the source of extracting data. Transformation refers to the transformation of facial emotion data into an emotional swatch. Implantation is the replacement of the color on the original art painting with the color of the emotional swatch of the user, and the resulting production of derivative images of the art painting is realized. Among them, extraction, transformation, and implantation are the key steps under this method, as shown in Figure 1.

### Extraction: Feature Extraction of Facial Expressions

Facial expression extraction can use image recognition technology. Image recognition technology is an important research category of artificial intelligence, and its recognition process is divided into image input, image preprocessing, image feature extraction, recognition result output, and label classification (Anwar et al., 2018). With the introduction of the concept of deep learning, the efficiency of image recognition technology has been greatly improved, and its application has become increasingly extensive. In image recognition technology, the most widely used is CNN (Krizhevsky et al., 2017). The model can convert an image into machine-understood data and process it by an algorithm so that the feature value can be extracted from the image data. Using CNN for facial and emotional recognition has been a major research topic in recent years, and many researchers have made improvements based on classic CNN to propose more accurate facial expression recognition methods for people. For example, Mollahosseini et al. (2016) combined AlexNet with the Google Net model to achieve better facial expression recognition. Lopes et al. (2017) combined some specific feature extraction methods with CNNs to improve the accuracy of expression recognition. Verma et al. (2019) proposed a network with branches of visual and facial identity that achieved a high recognition rate on the CK-plus dataset.

**Figure 2:**
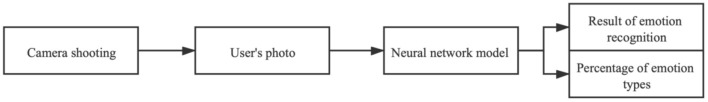
The process of extraction.

Due to the advantages of image recognition technology, in “extraction,” image recognition technology can be used to shoot users in real time through a camera so that the facial emotional characteristics of the user are extracted quickly and accurately. The main purpose of extraction is to convert the facial emotional characteristics of the user into computer data that the computer can understand. The system inputs photos of the user into the CNN model after certain processing and uses the model to obtain results composed of different kinds of facial emotional characteristics data. After normalization processing, the proportion data of various emotional categories are formed, and finally, the system transfers the data to the next process to complete the generation of an emotional palette, as shown in Figure 2.

### Implantation: The Transformation of Facial Expression Data into an Emotional Palette

**Figure 3:**
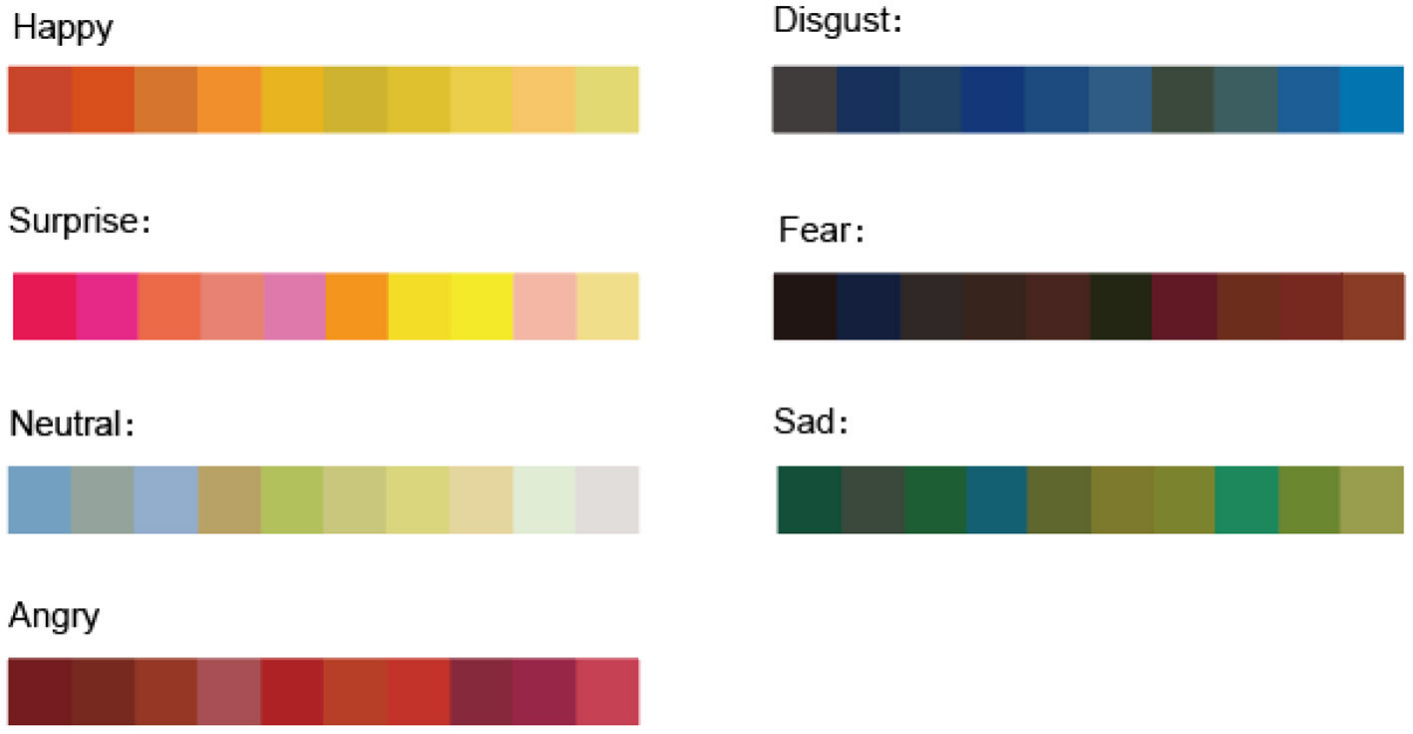
Emotional palette.

The main purpose of the “transformation” session is to form an emotional palette that is accurate to the individual user based on data extracted from facial emotional characteristics to facilitate the replacement of the original color of the art in the next steps. Taking Van Gogh as an example, this study classifies colors as emotional symbols by reference to the research of symbolism and psychology of colors, focusing on Van Gogh (Cela-Conde et al., 2010). In color habits of Van Gogh, yellow or orange colors represent positive and happy; saturated yellows and peaches represent surprise and joy; light colors with lower saturation, such as sky blue, pale green, and goose yellow, represent calm and leisure; red represents anxiety and anger; heavy green or blue green represents despair and sadness; blue represents a conflict between disgust and inner desire; and black and crimson represent depression and fear. According to the above color law, each type of facial emotion will be extracted to correspond to the color system of related emotion expression. Each color system has 10 colors, and finally, a complete type of facial emotion to color mapping will be constructed. After constructing the relationship between emotion data and color mapping, the individual emotion data are matched with the specific color by an algorithm, and the emotional palette is formed accurately to the individual to ensure the diversity and richness of the emotional palette. The emotional palette is shown in Figure 3.

### Implantation: Replacement of the Color of the Original Painting by an Emotional Palette

**Figure 4:**
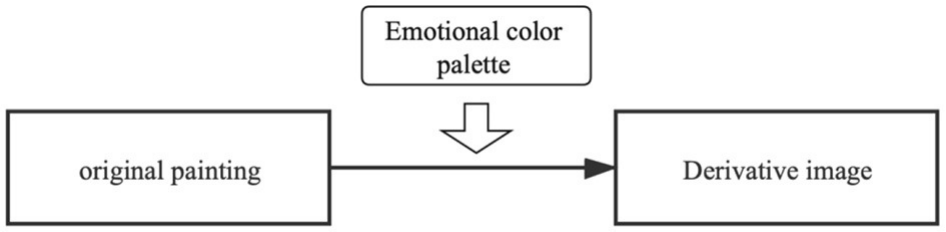
The color replacement process.

**Figure 5:**
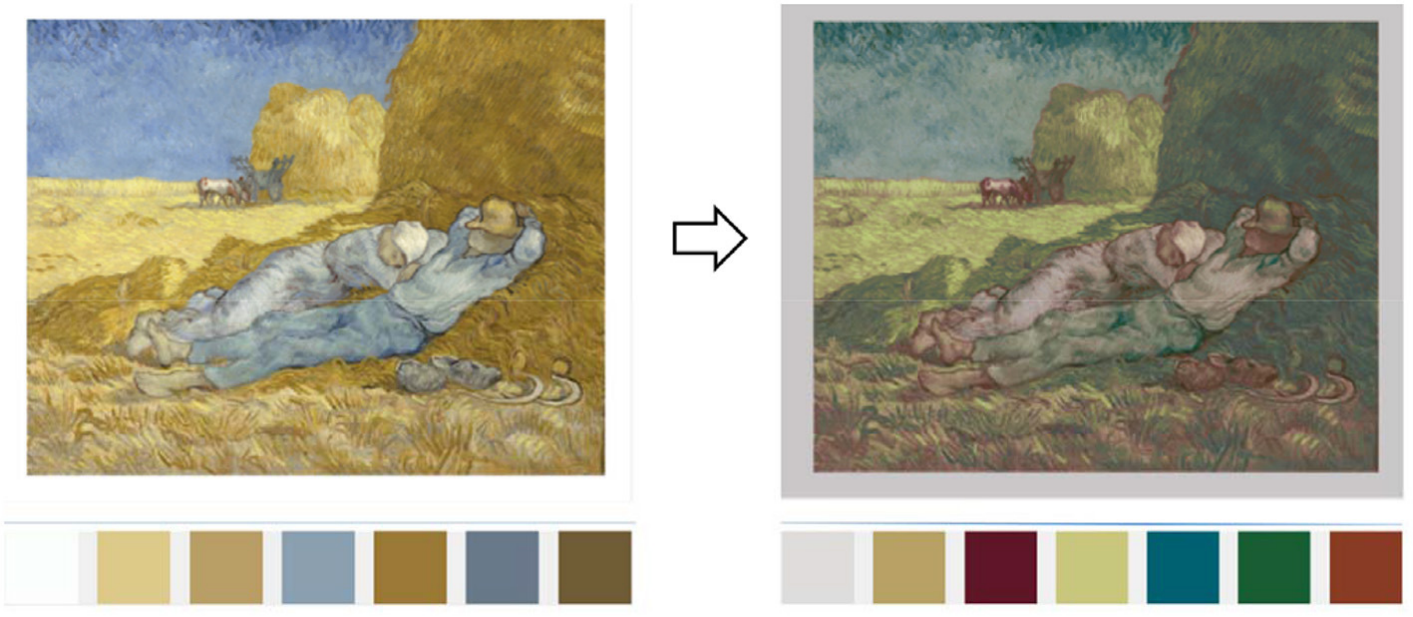
The transformation of original painting to the derivative image.

The main purpose of implantation is to automatically replace the color of the original painting with an emotional palette and to preserve the main features of the original painting, as shown in Figures 4, 5. To date, many scholars have worked out ways to recolor the image, such as Li et al. (2015), who used geodesic line spacing to complete the recoloring of the image while ensuring color coordination throughout the image in 2015. In the same year, Chang et al. (2015) proposed an interactive color replacement system based on palettes that can visualize images, and Cho et al. (2017) trained PaletteNet using a framework of deep neural networks, dramatically reducing the time to recolor in 2017. Replacing the color of the original painting with an emotional palette that is accurate to the individual user and using the “color” in the derived image as a personalized variable manipulated by the emotional characteristics of different users can realize the differentiated design of thousands of people and establish the emotional connection between the derived image and the user.

## Simulation Experiment

**Figure 6:**
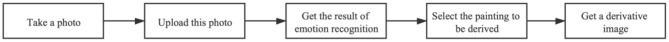
The user operation flow chart.

**Figure 7:**
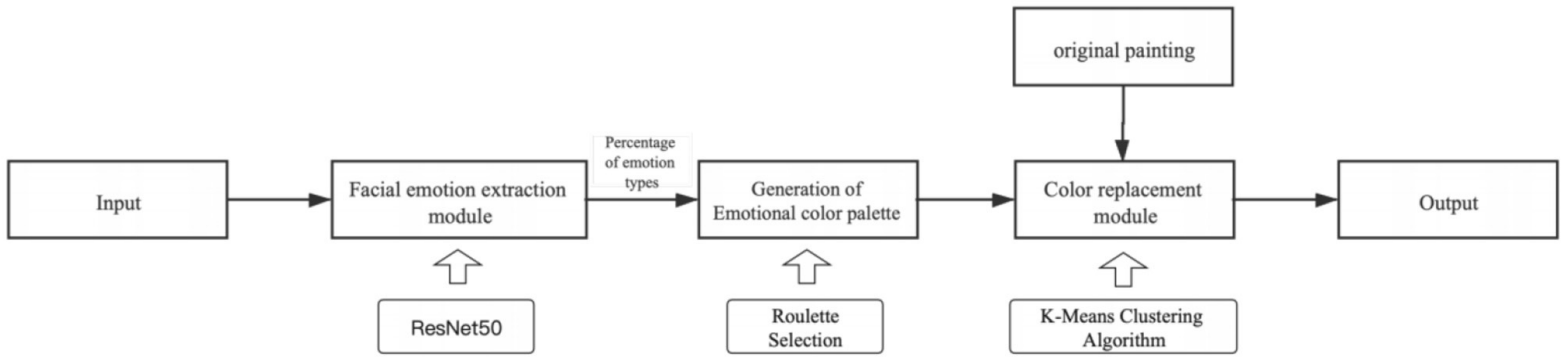
The System flow chart.

Based on paintings of Van Gogh, this study combines the traditional method with deep learning to construct a complete personalized derivative image design system, which is driven by emotion analysis for simulation testing, to verify the feasibility of the new method. The system is divided into three main modules, namely, the facial emotional feature extraction module, the emotional palette generation module, and the original color replacement module. The actions of the user are as follows: first, the user needs to make an expression in front of a camera to shoot. Then, from the images provided to the user by the system, the user selects the original painting that will be derived. Finally, the system changes the color of the original image by emotional analysis and image processing and generates the corresponding image derivatives, as shown in Figure 6. Correspondingly, the operating process of the system is as follows: first, the system reads the photos taken by the camera and passes them into the facial emotional feature extraction module to finish the transformation of facial emotional characteristics into computer data; then, the extracted data are fed into the emotional palette generation module to create a corresponding emotional palette, and finally, the emotional palette is entered into the color replacement module to replace the original color, as shown in Figure 7.

### Module 1: The Facial Emotional Feature Extraction Module Based on ResNet50

**Figure 8:**
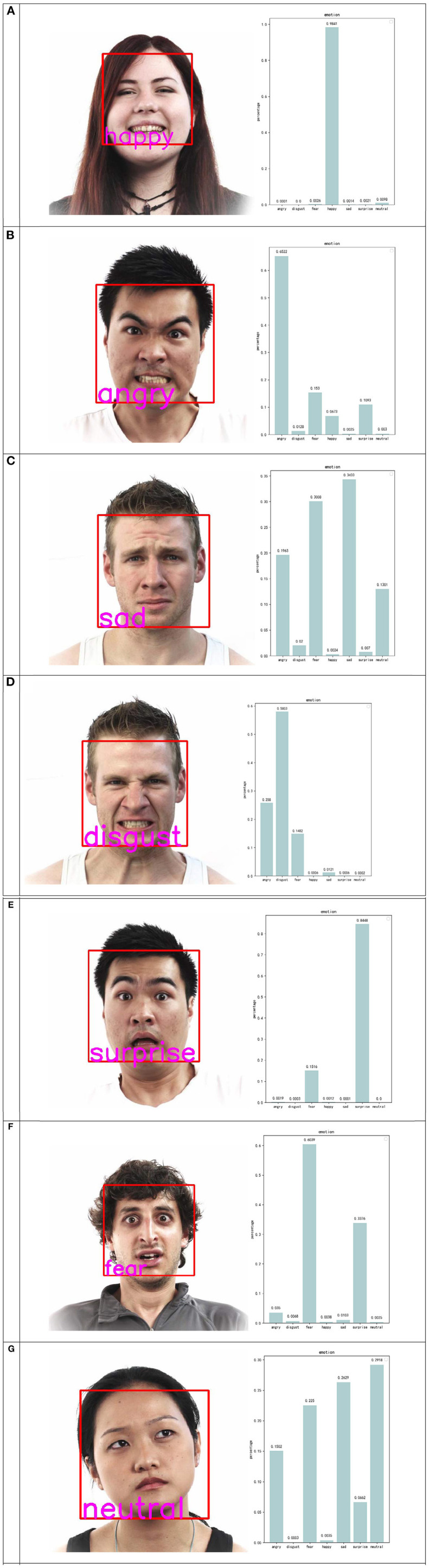
A visualization chart of facial emotional photos: **(A)** happy, **(B)** angry, **(C)** sad, **(D)** disgust, **(E)** surprise, **(F)** fear, and **(G)** neutral. Facial images are from the public dataset fer2013, which can be downloaded at https://www.kaggle.com/deadskull7/fer2013.

The main purpose of facial emotion extraction is to transform the emotions of the user into computer-understandable data through recognition and extraction to push the design process and influence the design results. In this study, the CNN model is constructed using Keras in the back end of Tenserflow. In the selection of the data set, both the training set and the test set used Kaggle's fer2013 dataset, a large data set that contains all the facial expressions of people, containing 28,000 training photos and 30,000 test photos, each of which is stored at 48 × 48 pixels. To make the extraction of facial emotional characteristic data more accurate, this study uses three neural network models, classical CNN (Krizhevsky et al., 2017), VGG16 (Dubey and Jain, 2020), and ResNet50 (He et al., 2016), and finally chooses ResNet50, whose accuracy is 70.89%, ranking among the top three. In the extraction of facial emotional characteristics, the more critical step is to classify emotions. Therefore, in the process of building neural networks, the more important step is to store facial expressions as values. In this process, Keras generates an output array that contains seven different emoticon scores: happy, angry, sad, disgust, neutral, surprise, and fear. After finishing the training, the system will see the category whose value is largest as the result of emotion recognition. After normalizing these emoticon scores into proportional values in a certain way, the system outputs a visualization chart of facial emotional data and a photo with the recognition result in the form of a bar chart, as shown in Figure 8.

Although the module solves the problem of facial emotional feature extraction, there are still some problems. First, in the extraction, the accuracy of the available model is not high enough, especially for the recognition of microexpressions, and is prone to misjudgment. For example, when the corners of the mouth of a person are slightly sagged, the brow is slightly wrinkled, and the eyes are full of grievances, the result of the system judgment is “calm.” Obviously, this is not in line with the judgment of a human, in which the result should be “sad.” Therefore, in the follow-up study, we can improve this by expanding the data set or adjusting the neural network model to improve the accuracy of the emotion analysis and realize the precise recognition of the microexpression of the characters. In addition, the existing facial emotion extraction methods can only analyze static pictures and cannot perform emotion recognition in dynamic images. It is precisely because of this limit that users are required to shoot facial expressions in front of a fixed camera. Such restrictive behavior makes the interaction stiff and not natural enough, and timely feedback on emotion from the users cannot be obtained when they are enjoying the exhibition. Therefore, in subsequent studies, how to extract data from a timely camera video and how to use these data in the design process are the key issues to optimize the interaction.

### Module 2: The Emotional Palette Generation Module Based on Roulette Wheel Selection

Based on the established facial emotion data and color mapping relationship, this study uses roulette wheel selection (Lipowski and Lipowska, 2012) to complete the matching of emotion data and specific colors to form an individual emotional color palette. Roulette wheel selection is often used in genetic algorithms to increase the diversity of individual generations. The principle is similar to a turntable for a lottery, in which seven regions are divided according to the type of emotion, each corresponding to an emotional color. The proportion of the region is determined by the proportion of emotion types, and a higher proportion of emotion will have a higher probability of being selected. The pointer of the turntable rotates randomly, and where the pointer stops, a color is randomly selected from the 10 colors of the emotional color. To avoid multiple selections of the same color, the selected color will be rejected after the selection. The emotional palette chosen in this study is composed of seven colors. To make the final formation of the palette, seven emotional corresponding color classes are selected. The system drives the pointer to rotate seven times through roulette wheel selection, selecting an emotion class at a time and randomly selecting a specific color with equal probability in this class. The system follows the above process seven times to generate an emotional palette, which can make the color class corresponding to the larger emotion species have more opportunities to be selected, ensure the consistency of the resulting emotional palette tone, and clearly reflect the facial emotional characteristics in the photo of the user.

**Figure 9:**
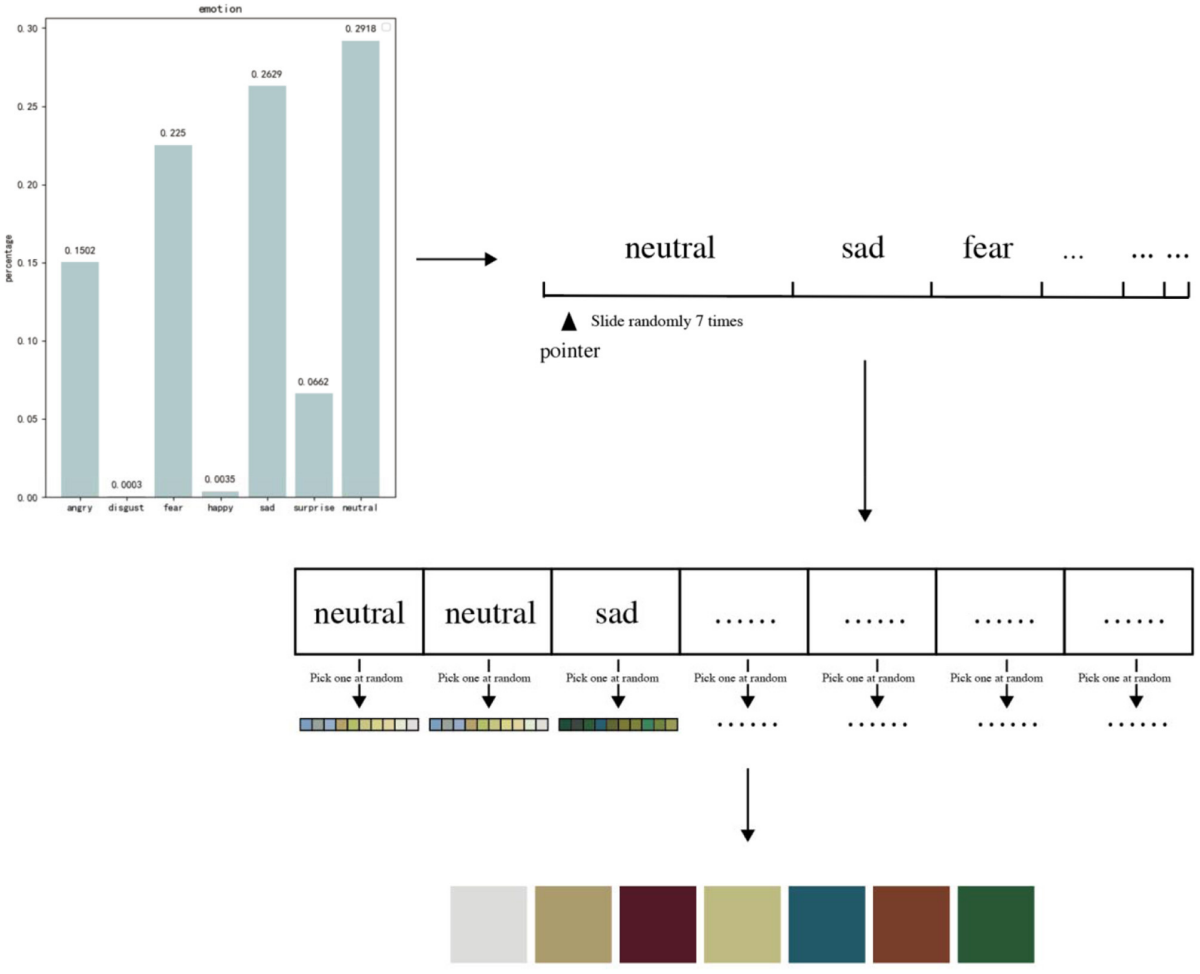
Emotional palette transformation.

In the process, the roulette wheel selection integrates the probability graph, which is originally fan-distributed, into a straight line, divides the interval (0, 1) into seven segments according to their respective emotional classes, and then generates a random floating point located within the interval (0, 1). The system selects the emotion type through pointer selection, randomly selects a color from the color class corresponding to the emotion type to add to the emotional palette, and then deletes the color from the color library to avoid repeatedly selecting the same color. After repeating the above process seven times, the system chooses seven colors to form the final emotional palette. This method used in this study can not only ensure that users generate completely different emotional palettes for each shot but also avoid the situation in which users with similar emotions have emotional palettes. This method can increase the randomness of palettes. In addition, in the testing process, we found that the facial emotion data occasionally have extreme situations. For example, when a person is laughing, the emotion extraction system will give “happy” a very high value. When transferred into proportional data, it occupies a large area, and the proportion of other emotions is compressed into a small amount. Since the proportion of emotion is directly related to the emotional color palette, to avoid the problem that the final generated color palette is all from the same color class and the color tone of the new image is too single after color replacement, this study adds the “if” condition to the roulette wheel selection. When the probability of a single type of color class selection does not exceed 50%, the tone of the new image is relatively balanced. Therefore, the conditional discrimination statement detects the probability of each type of color class, and when the probability of a color class is more than 50%, the conditional discrimination statement limits it to 50%, with the remaining 50% divided equally by other classes according to their proportions, as shown in Figure 9.

Here, we present the code of our adopted model in Algorithm 1.

**Algorithm 1:** The adopted model in this study

begin
Number the emotions in turn *E*~[1, 7],
Divide the interval (0, 1) into 7 parts, in order of probability, and record it as interval 1-7,
*N*←1
While(*N*>7) do
*P*=random(0,1) //*P* is the number of random floats generated within the interval(0, 1).
if P is within the interval *i* // *i*~[1, 7]
then *E*←*i*
end if
Randomly select a color from Mood *E* to add to the palette
*N*←*N*+1
end
end

However, as far as the expected effect is concerned, the method of generating emotional palettes proposed in this study meets the need to match emotion and color, but there are still some shortcomings. The main problem is that, although the method fully satisfies the randomness of palette generation, it neglects the harmony of color matching. In these randomly generated palette, because of the lack of boundary between color and color, it will lead to a great difference between the result of color replacement and the expected effect. The appearance of color can not meet the aesthetic standard, which will affect the user's aesthetic experience. Therefore, in follow-up research, it is necessary to establish a mathematical model according to the requirements of color harmony and aesthetic standards, and the system needs to limit the original algorithm or find a new and more suitable algorithm.

### Module 3: Color Replacement Module Based on the K-means Clustering Algorithm

**Figure 10:**
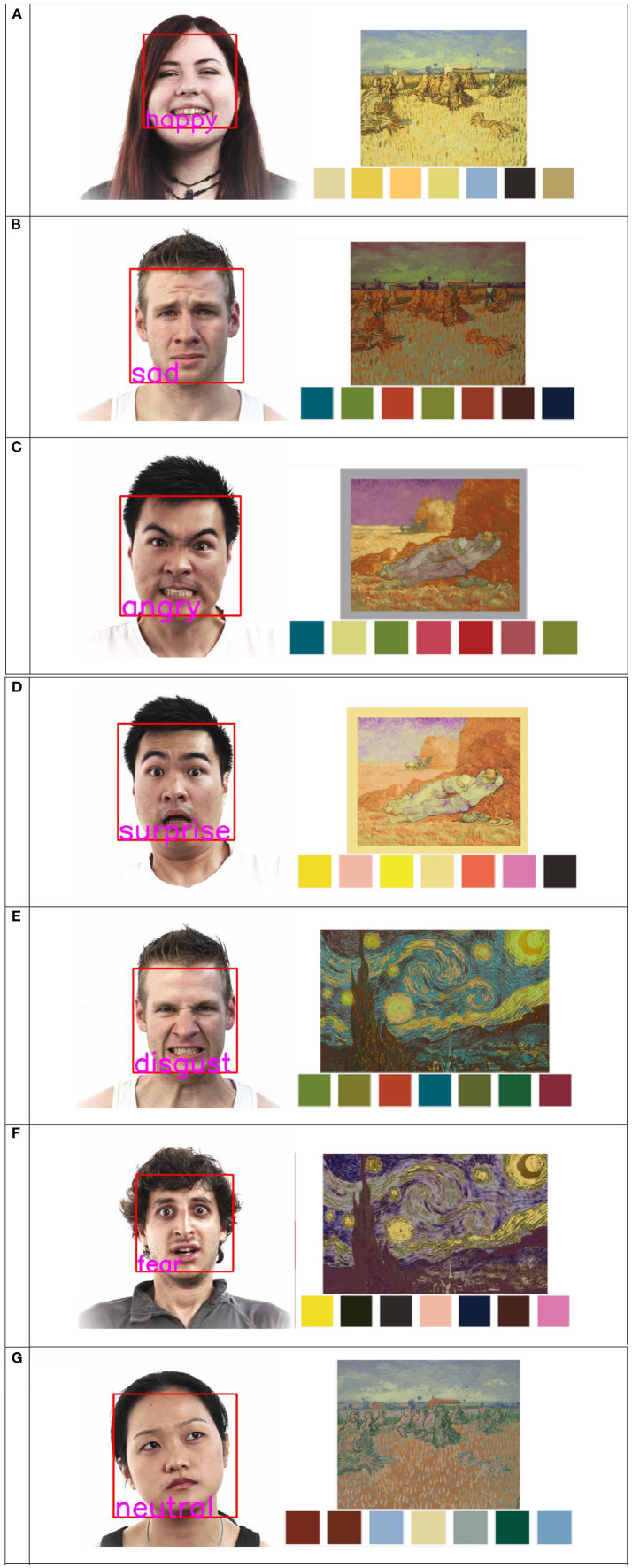
A derivative picture of **(A)** happy, **(B)** sad, **(C)** angry, **(D)** surprise, **(E)** disgust, **(F)** fear, and **(G)** neutral. Facial images are from the public dataset fer2013, which can be downloaded at https://www.kaggle.com/deadskull7/fer2013.

The purpose of the color replacement module is to replace the original palette with the emotional palette to complete the color transformation of the original image. As the modules are built, this study refers to the project of the team of Kanyaraasi, Palette-based Photo Recolor (Chang et al., 2015), to build the color replacement system. The image recoloring system proposed by the Kanyaraasi team has a complete image recoloring interface that allows users to replace the color of palettes one by one by mouse interaction, depending on their personal preferences. Based on this system, this study proposes an image recoloring system driven by an emotional palette corresponding to facial emotional characteristics, which realizes the full automation of the process. The color transformation system is based on the K-means clustering algorithm, which extracts the palette of the original painting and realizes the color transformation processing of the image. To improve the clustering efficiency of the K-means algorithm, first, the three channels of the image RGB are normalized, and then, based on the statistical results of the image histogram, the channels are divided into 16 sets of histogram columns. Each histogram column contains several pixels, and then 16 × 16 × 16 statistical histogram columns can be obtained. The Lab spatial color mean of the pixels in each statistical hist bar is calculated as the cluster color center of each statistical hist square bar, and then, the 16 × 16 × 16 cluster color centers are clustered using the K-means clustering algorithm. The cluster center is divided into seven categories. The color of each cluster center is a kind of palette, and then, the original image of seven palettes is extracted. Then, with the emotional swatch proposed above, according to a specific method of one-to-one corresponding processing, the original palette is replaced to achieve the color conversion of the image. Eventually, the system outputs the image with the emotional characteristics of the user as a derived image to the user, and the result of color substitution is shown in Figure 10.

Although the existing color replacement module has completed the color replacement of the original image, there are still some problems. The main problem with the replacement is that the derived image cannot retain the light and dark layers of the original color distribution. When the original art is transformed into a derivative image, the problem will cause the color layer of the image to be greatly weakened or become insufficiently harmonious. Moreover, it does not conform to the context of the picture, and the meaning expressed by the picture is the opposite. Therefore, in the follow-up research process, researchers need to start from the painting semantics and color light and dark structures in the process of color replacement to limit the brightness of the picture so that the derivative image more closely fits the original rather than only from the picture color ratio of the area to make a simple color replacement.

## Design of the Personalized System Based on Emotion Analysis

Through the above research, the new design method is implementable in combination with new technology, and the intervention of new technology can effectively solve the existing problems in personalized design. Even so, there are two issues in the existing methodology that can be improved. First, in the existing personalized design process, the solution proposed in this study focuses on the user of the characteristic extraction stage. In other stages, using different technologies to achieve more personalized user needs of the design is still worth exploring; it is also a direction worth studying later.

In addition, in the construction of the personalized design simulation system in this study, the main technology is machine learning, and other computer technologies are secondary technologies. This phenomenon verifies that the development of new technology, especially the wide application of machine learning, can promote the change of design methods and provide the possibility for the emergence of new design paradigms. As far as the entry point of this study, i.e., personalized design, there are two possible changes in the design method: one is the function changes of the designer in the design process, and the other is that the connection between user characteristics and personalized elements is becoming increasingly diverse and complex.

### Functional Changes in the Design Process of a Designer

**Figure 11:**
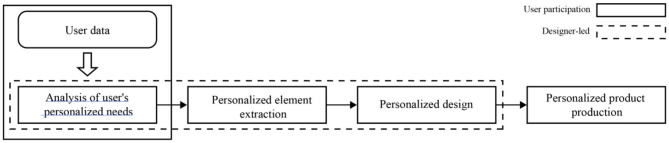
Traditional personalized design approach and role engagement.

**Figure 12:**
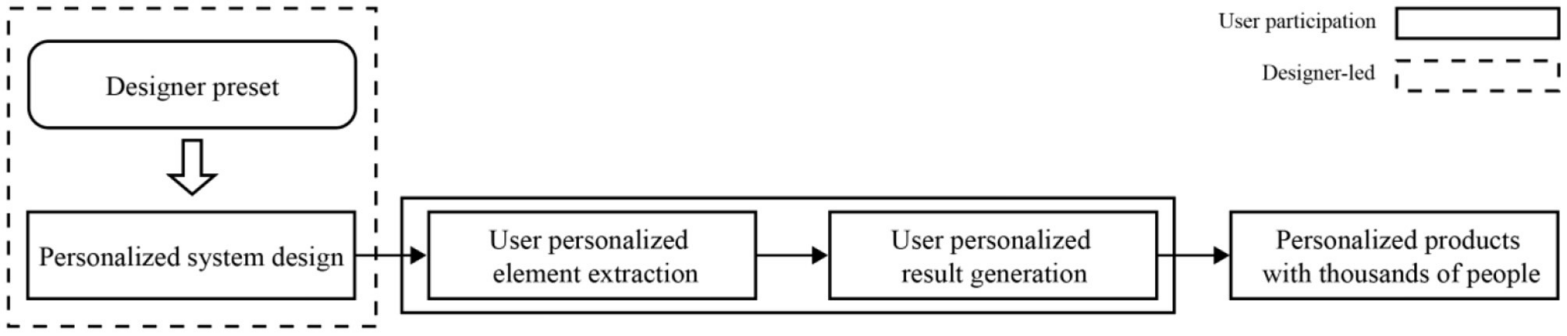
Combining new technology to complete the design of the personalized system.

Traditional personalized products are roughly divided into four links from design to production: user personalized demand analysis, personalized element extraction, personalized design, and personalized product production. In this design process, the designer occupies a dominant position, and user data are only involved in the analysis of the individual needs of the user in the early stage. In the subsequent design links, the designer controls the subsequent process as the lead, as shown in Figure 11. To better serve users and meet their emotional needs, future intelligent personalized design methods need to allow more user data to participate in the entire design process, and the entire design process should be more dominated by users or user data. The sense of participation of the user should be enhanced to achieve true participatory consumption. The role of the designer in this process should be a designer of the personalized design system. Its main task is to combine new technology to complete the design of the personalized system and the interactive process of user participation in the personalized design, as shown in Figure 12. After finishing these tasks, the designer transfers the leading power to the user, allowing the personalized system and the user to cooperate with each other to complete the design process together and allowing the user to complete the customization of personalized products according to their personal preferences and realize the real sense of “thousands of needs” design.

### The Connection Between User Characteristics and Personalized Elements

**Figure 13:**
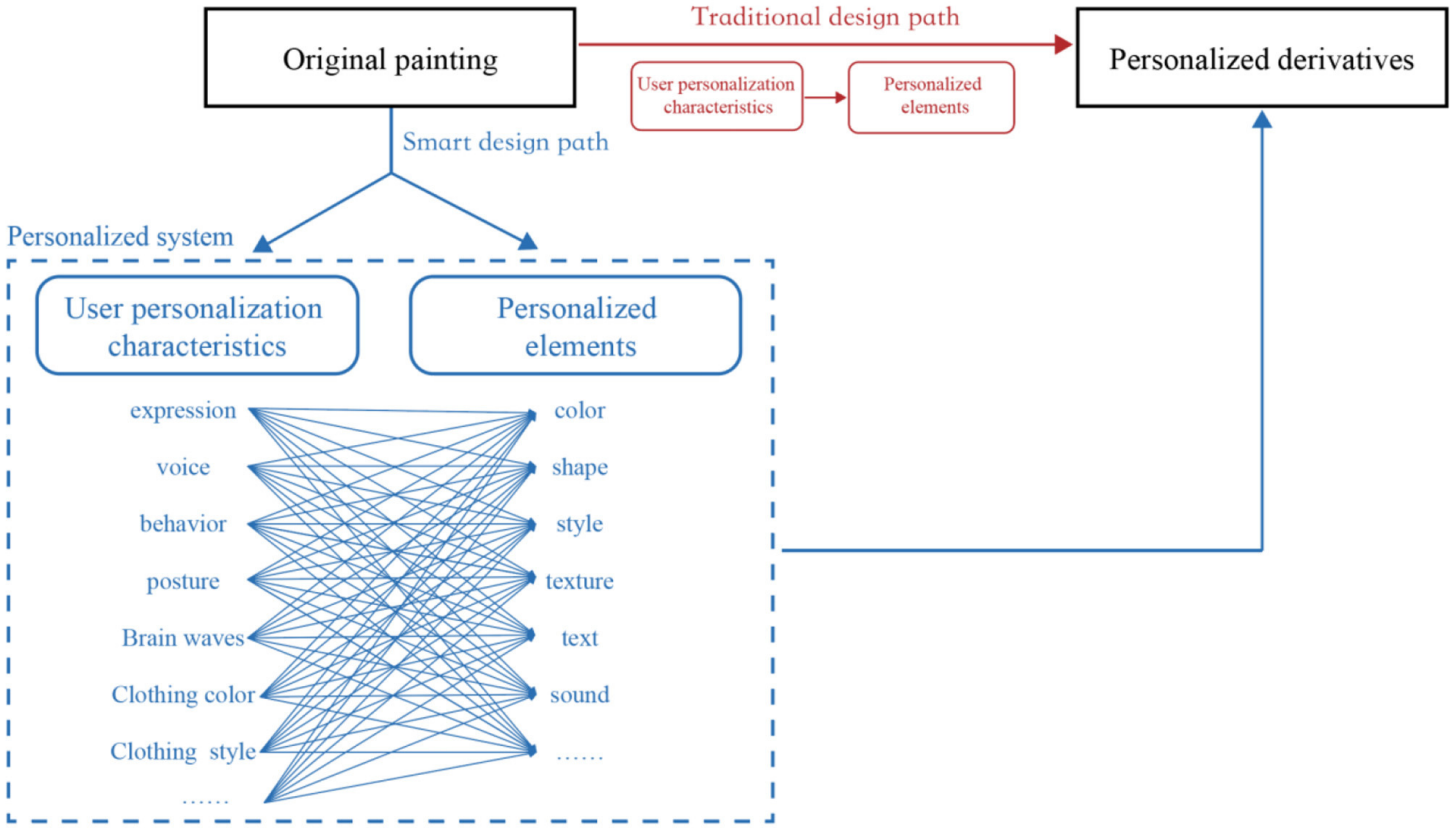
The relationship between user emotional characteristics and individual elements.

In traditional personalized design, the source of user characteristics mostly comes from user research; designers need to extract user characteristics and preferences from the results and, then, through certain means, transform the characteristics and preferences of the user into personalized elements and integrate them into personalized products, which is also the key step to establish personalized product user emotional connections. However, due to the limited amount of data and the limited ability of designers to process data, it is difficult to meet the needs of all when extracting user characteristics and building user emotional connections. However, with the development of big data and artificial intelligence and with the help of intelligent technology, an increasing number of user features can be mined and extracted. In addition to the advantages of “many quantities,” intelligent technology with the advantages of user characteristic extraction also has the advantages of high speed and high precision. With such technical support, the emotional feature extraction of the user can be multidimensional, fast, and precise so that the extraction of user characteristics can be more comprehensive and more diverse to find more dimensions that can serve the personalized design. As far as the project studied in this study is concerned, in the future design, more kinds of emotional characteristics of users can be extracted, and the elements used to extract the emotional characteristics of the user are the facial expressions of the user. Facial expression is the materialized embodiment of the emotion and expression of the user of the direct emotion of the user. In addition, it can also be extracted by extracting the voice, behavior, brain waves, and other elements of the user that directly reflect emotional characteristics to form an emotional connection with personalized products or by extracting color matching, wearing style, and other elements that indirectly reflect emotional elements to establish the meaning of the user and personalized products. As the number of emotional feature elements that can be extracted increases, so does the number of personalized elements in the corresponding product, while each element representing the emotional characteristics of the user can be associated with different personalized elements, such as shape, style, and texture, in addition to color. Therefore, compared with the relationship between the emotional characteristic elements and the personalized elements of the user in the past, the relationship between the two is more complex and diverse in intelligent personalized design in the future, as shown in Figure 13.

## Conclusion

This study proposes a new method of art image derivative design based on emotion analysis. First, the significance of personalization is analyzed in the design of art derivatives. Then, the problems existing in the personalized design method are pointed out, the new needs of users in the background of intelligent technology are analyzed, new design ideas combined with new technology are put forward, the main technical principles of the technology are analyzed, and practical solutions are proposed to explore the new design paradigm. Finally, by constructing a personalized design system of image derivatives driven by user facial emotion data based on paintings of Van Gogh, this study verified the feasibility of the solution. It was found that innovation of the design paradigm is imperative. It is significant to change the original design paradigm and introduce new ones, which can make the design better combined with current new technology and better serve the needs of the public.

There are also some problems that can be improved. The facial emotion feature extraction module mainly uses ResNet50 to extract the facial emotion data of the user. Its shortcomings are that the accuracy of this module is not high enough and extracting emotion data from video and processing them cannot be achieved; the emotion color palette generation module uses roulette wheel selection to realize the generation of emotional palettes, but its disadvantage is that the color matching of the generated palettes cannot fully meet the aesthetic standards. The original color replacement module uses the K-means clustering algorithm to achieve the color replacement of the original image based on the emotional palette. The disadvantage is that it cannot completely retain the beauty of the light and dark levels of the original color.

## Data Availability Statement

Publicly available datasets were analyzed in this study. This data can be found here: https://www.kaggle.com/deadskull7/fer2013.

## Ethics Statement

Written informed consent was obtained from the individual(s) for the publication of any potentially identifiable images or data included in this article.

## Author Contributions

YD, JZ, and XG developed the theoretical framework and the model in this study and drafted the manuscript. YD implemented the algorithm and performed experiments and result analysis. All authors contributed to the article and approved the submitted version.

## Conflict of Interest

The authors declare that the research was conducted in the absence of any commercial or financial relationships that could be construed as a potential conflict of interest.
